# Assessing Schmallenberg Virus Disease in Sardinia (Italy) After the First Epidemic Episode in 2012

**DOI:** 10.3390/pathogens14040349

**Published:** 2025-04-04

**Authors:** Cipriano Foxi, Davide Pintus, Susanna Zinellu, Simonetta Macciocu, Pier Paolo Angioi, Anna Maria Sechi, Mariangela Stefania Fiori, Anna Ladu, Graziella Puggioni, Stefano Denti, Maria Luisa Sanna, Maria Paola Madrau, Giuseppe Satta, Annalisa Oggiano, Ciriaco Ligios, Silvia Dei Giudici

**Affiliations:** Department of Animal Health, Istituto Zooprofilattico Sperimentale della Sardegna, 07100 Sassari, Italy; cipriano.foxi@izs-sardegna.it (C.F.); davide.pintus@izs-sardegna.it (D.P.); susanna.zinellu@izs-sardegna.it (S.Z.); simona.macciocu@izs-sardegna.it (S.M.); pierpaolo.angioi@izs-sardegna.it (P.P.A.); annamaria.sechi@izs-sardegna.it (A.M.S.); mariangela.fiori@izs-sardegna.it (M.S.F.); anna.ladu@izs-sardegna.it (A.L.); graziella.puggioni@izs-sardegna.it (G.P.); stefano.denti@izs-sardegna.it (S.D.); marialuisa.sanna@izs-sardegna.it (M.L.S.); paola.madrau@izs-sardegna.it (M.P.M.); giuseppe.satta@izs-sardegna.it (G.S.); annalisa.oggiano@izs-sardegna.it (A.O.); ciriaco.ligios@izs-sardegna.it (C.L.)

**Keywords:** Schmallenberg virus, *Culicoides*, phylogenetic analysis

## Abstract

Schmallenberg virus (SBV), an *Orthobunyavirus* transmitted by *Culicoides*, causes congenital malformations and mild symptoms, such as fever, reduced appetite, decreased milk production, and occasional diarrhea, in ruminants. First detected in Central Europe in 2011, SBV spread across the continent, reaching Sardinia (Italy) in late 2012. This study evaluates the occurrence of SBV infections in Sardinian sheep from 2013 to 2024 by anatomo-pathological, virological, serological, and entomological data. The results suggest the presence of SBV infections in a continuous enzootic status over the years, without the cyclic waves observed in other countries, likely due to the unique sheep breeding management in Sardinia. Seroprevalence rates in the years 2022 and 2024 varied between 16.40% (C.I. = 12.28–20.52) and 21.53% (C.I. = 17.15–25.91) without significant differences between the two years analyzed. SBV was predominantly detected in *C. imicola* and *C. newsteadi* populations, while *C. cataneii* and *C. sahariensis* were identified as potential new vectors. Additionally, S- and M-segment sequences were obtained from two SBV isolates, S-sequences from a sample detected in 2020, and 21 archived cDNA samples from 2012. The S-segments showed high similarity among themselves and the reference strains, while the M sequences were significantly different, although potential artifacts from fetal samples must be considered. Overall, the results suggest widespread enzootic SBV circulation in Sardinia over the past decade, with a very low frequency of malformations in newly born sheep offspring.

## 1. Introduction

Schmallenberg virus (SBV) is an arbovirus belonging to the *Orthobunyavirus* genus within the *Peribunyaviridae* family and part of the Simbu serogroup. It was initially isolated in Central and Western Europe from adult dairy cows exhibiting mild symptoms, including diarrhea, fever and reduced milk yield [1]. Subsequently, SBV has been reported to affect other domestic and wild ruminants, such as sheep [2], goat [3], deer [4], and water buffalo [5]. A notable pathological feature of SBV is its ability to pass the placenta barrier in pregnant animals, leading to early-stage abortion and malformations in stillborn offspring [3,6]. While the direct transmission of SBV is considered unlikely, biting midges of the *Culicoides* genus play a fundamental role in transmitting SBV, thus causing seasonal spread in late summer and autumn. SBV RNA has been detected in various *Culicoides* species in the Netherlands, Belgium, France, Poland, and Italy [7,8,9,10,11].

The genome of RNA SBV consists of three segments: a large segment (L) encoding viral RNA, a medium segment (M) encoding structural (Gn, Gc) and non-structural (NSm) proteins, and a small segment (S) encoding a non-structural protein (NSs) and the nucleocapsid protein [12]. High genetic variability, including deletion and point mutations, has been observed in the 5′-terminal part of the M-segment, which encodes the SBV envelope Gc protein, a major target of neutralizing antibodies [13].

However, M-segment sequencing data are often biased, especially in sheep, as most viral genomes were sequenced from tissue samples of malformed fetuses rather than from blood samples of viremic hosts [13,14]. This is due to the brief viremic phase in the affected host, so caution is required when interpreting the implications of mutations in the M-segment. SBV spread rapidly in Europe during 2011, driven by the naive status of the susceptible ruminant population and the absence of effective control and prevention measures [15]. In the following years, SBV was still reported in several European countries, although with limited circulation and sporadic clinical cases occurring at intervals of 3–4 years [16,17]. In late 2012 and early 2013, SBV infections were widely reported in Sardinia [18]. During an epidemic of arboviral diseases, various environmental, climatic, entomological, and management factors can significantly influence disease occurrence, potentially imparting the disease with unique characteristics in different regions. Thus, SBV’s epidemiology and epidemic progression may vary geographically, necessitating region-specific investigations to design tailored control and surveillance strategies and to assess potential economic losses [19].

In this study, we evaluate the occurrence of SBV infections, particularly within the Sardinian ovine population, following the first epidemic wave in late 2012. This assessment is based on a decade of anatomo-pathological, virological, serological, and entomological data. Furthermore, to gather baseline information on the genetic characteristics of the SBV strain circulating in Sardinia, we analyzed the S and M-segment sequences from two isolates and the S-segment of both a positive sample collected in 2020 and twenty-one archived cDNA samples collected from malformed fetuses during the first epidemic wave in 2012.

## 2. Materials and Methods

### 2.1. Retrospective Study

A retrospective survey was conducted in Sardinia, analyzing data from diagnostic activities on pathological conditions affecting fetuses and newborn sheep, cattle, and goats at the Istituto Zooprofilattico Sperimentale of Sardinia, covering the period from January 2013 to December 2023. During this time, 340 fetuses and newborn ruminants from 213 farms were examined using anatomo-pathological, serological, and virological diagnostic methods. Specifically, we focused on the results of anatomo-pathological and virological examinations conducted on farms where SBV infection was suspected. SBV cases were confirmed based on the presence of one of the following three criteria: (i) detection of viral RNA, (ii) viral isolation in cell culture, and (iii) the presence of typical malformations in newborn animals, such as brachygnathia, arthrogryposis, curvature of the vertebral spine, hydrocephalus, and central nervous system hypoplasia (Figure S1) [20].

### 2.2. Serological Investigation

Specific SBV IgG detection was carried out in Sardinia examining serum samples collected during the period 2013 to 2023 from sheep (n = 421), cattle (n = 0.174), and goat (n = 65) farms, in which conditions of infertility due to early embryonic death, abortion, and malformed newborns were reported. In addition, serum samples randomly collected from the sheep population of Sardinia in the years 2022 (n = 311 animals) and 2024 (n = 339 animals) were analyzed. Given the literature data [5], the sample size was calculated considering an expected prevalence of 40%, a maximum accepted error of 5%, and a confidence interval of 95%. SBV antibodies detection was performed by ID Screen^®^ Schmallenberg virus Competition Multi-species (Innovative diagnostics, ID.vet), which was used according to the manufacturer’s instructions.

A Chi-squared test (χ^2^) was used to test the difference in the SBV seroprevalence in 2022 and 2024. Statistical analyses were carried out using SPSS v.21 software with *p* value < 0.05 regarded as statistically significant.

### 2.3. Real-Time PCR (RT-PCR) Analysis

Tissue samples from fetuses or malformed lambs, kids, and calves collected between 2013 and 2023 (see the retrospective study paragraph) were analyzed for SBV genome detection. The following organs and body fluids were tested: spleen, cerebrum, kidney, thyme, umbilical cord, and placenta. Each tissue sample was homogenized in phosphate-buffered saline (PBS) at 10% (*w*/*v*) and centrifuged at 1000× *g* for 10 min.

Two hundred microliters of clarified tissue homogenate, body fluids, or whole blood samples were processed for RNA extraction using the MagMax core and the automatic MagMax expression 96 instrument (Applied Biosystems, Foster City, CA, USA), according to the manufacturer’s instructions. All the samples were screened for the presence of SBV by qualitative real-time RT-PCR (qRT-PCR) using the AgPath-ID One-Step RT-PCR Kit (Applied Biosystems, Foster City, CA, USA). A duplex assay was applied to detect the SBV genome combined with a β-actine housekeeping gene system using primers previously described [20,21]. Briefly, the master mix consisted of 12.5 µL of 2× buffer, 1.0 µL of enzyme mix, 0.8 µM of SBV primers, 0.15 µM of SBV probe [20], 0.2 µM of β-actine primers, 0.1 µM of β-actine probe [21] and 5 µL RNA template in a total volume of 25 µL. The thermocycling profile was established as follows: 10 min at 45 °C, 10 min at 95 °C, followed by 42 cycles of 15 s at 95 °C, 20 s at 55 °C and 30 s at 72 °C.

For SBV RNA detection in the pool of midges, samples were mechanically homogenized in 0.2 mL of antibiotic-treated PBS (400 U/mL Penicillin/Streptomycin, 200 μg/mL Gentamicin, 2.5 μg/mL Amphotericin B). One hundred microliters of insect homogenate were spiked with the EGFP plasmid [20] as an internal control. Total RNA was extracted using the MagMax core and the automatic MagMax expression 96 instrument (Applied Biosystems, CA, USA), according to the manufacturer’s instructions. SBV RNA detection was performed as previously described [20] by using the AgPath-ID One Step RT-PCR kit (Life Technologies, Carlsbad, CA, USA) in a duplex RT-PCR combining primers and probe for the SBV S-segment with those for the EGFP plasmid.

### 2.4. Isolation of SBV in Cell Cultures

Cell culture virus isolation was carried out on tissue samples from five newborn lambs collected in 2020–2021 from three affected farms in central and northern Sardinia and six insect pool samples of *Culicoides imicola*. All samples previously tested positive for SBV qRT-PCR with Ct values ≤ 30. VERO cells (African Green Monkey Kidney) and BHK21 cells (Baby Hamster Kidney clone 13) were provided by IZSLER-BVR—Brescia, Italy. The one-day-old cell cultures, 80–90% confluent, were grown in T25 cell culture flasks according to standard cell culture techniques. Tissue samples and insect pool samples were mechanically homogenized with ceramic beads (OMNI Bead Ruptor) in 4.5 mL and 0.2 mL of 1× antibiotic-treated PBS, respectively. The homogenates were then incubated for 2 h at room temperature and centrifuged at 1000× *g* for 10 min [22]. The clarified supernatants (0.5 mL for tissues and 0.1 mL for insect pool samples) were inoculated onto the cells monolayers, and the flasks were incubated for 2 h at 37 °C 5% CO_2_. The inoculum was removed and replaced with 9 mL per flask of antibiotic-treated culture medium supplemented with 10% Fetal Calf Serum—FCS (Eagle’s Minimal Essential Medium-EMEM for VERO cells, Dulbecco’s Modified Eagle Medium-DMEM for BHK21 cells with 100 U/mL Streptomycin, 50 μg/mL Gentamicin, 0.25 μg/mL Amphotericin B). The flasks were incubated for 5–6 days and assessed daily by microscopic observation. The infected cell cultures were lysed by freezing and thawing, and 0.5 mL of the lysates were inoculated onto cells for the next passages. A total of three passages were performed, and the virus presence was assessed on the basis of lysis plaque formation and confirmed by SBV qRT-PCR.

### 2.5. Sanger Sequencing and Phylogenetic Analysis

The two SBV isolates obtained in this study, one positive sample detected in 2020 and 14 SBV-positive cDNA samples collected during the epidemic wave in 2012, were submitted to Sanger sequencing and phylogenetic analysis. cDNA was generated using the extracted RNA and random hexamer primers with superscript III reverse transcriptase (Thermo Fisher, Waltham, MA, USA), following the manufacturer’s instructions. The S and M genome segments were amplified using primers described in Table S1 combined with primers whose sequence was kindly provided by Dr. Martin Beer (FLI, Insel Riems, Germany). The master mix consisted of 10 µL of 10× buffer, 2.5 mM of MgCl_2_, 0.2 mM of dNTPs, 0.125 µL of Platinum Taq DNA Polymerase (Thermo Fisher, Waltham, MA, USA), 0.2 µM of primers and 2 µL of cDNA template in a total volume of 50 µL. The thermocycling profile was established as follows: 5 min at 95 °C, followed by 40 cycles of 15 s at 95 °C, 30 s at 58 °C, 60 s at 72 °C and a final extension of 10 min at 72 °C.

Sanger sequencing was performed on both strands on an ABI-PRISM 3500 Genetic Analyzer (Applied Biosystems, Waltham, MA, USA) with a DNA sequencing kit (dRhodamine Terminator Cycle Sequencing Ready Reaction; Applied Biosystems, Waltham, MA, USA). The sequences were assembled, edited, and aligned with all the S and M international sequences retrieved from GenBank (accessed on 19 June 2024) and translated using BioEdit software, version 7.0.0 [23]. BLAST nucleotide analysis (https://blast.ncbi.nlm.nih.gov) was performed to identify the best matching sequences in the GenBank database. Haplotypes of sequences were identified using DNASPv6 [24].

The S and M-segment datasets obtained, composed of 160 and 104 sequences, respectively, were checked for recombination by RDP, GENECONV, MaxChi, and 3Seq methods in the RDP5 software [25]. The evolutionary model that best fitted the data was selected using JmodelTest v.2.1.7 [26]. The phylogenetic signal was subjected to the likelihood mapping analysis with 10,000 random quartets in the TreePuzzle software v. 5.2 as already described [27]. Phylogenies were reconstructed in MEGA 7 [28] via the maximum likelihood and GTR+G+I model of nucleotide substitution. Statistical support for specific clades was obtained via 1000 bootstrap replicates.

### 2.6. Entomological Survey

Collections of *Culicoides* were made in accordance with the protocols of the Italian Reference Centre for Exotic Diseases (CESME, Istituto Zooprofilattico Sperimentale dell’Abruzzo e del Molise).

**Figure 1:**
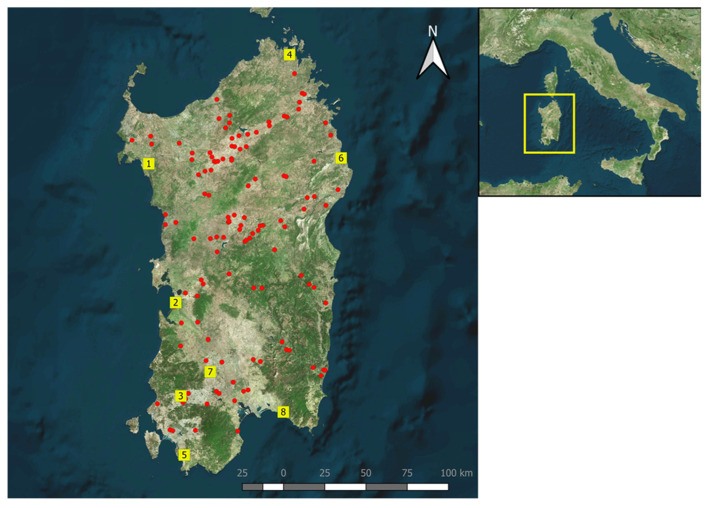
Map of Sardinia showing the distribution of the randomly selected sheep (red circles), which serologically were SBV positive. Yellow squares indicate *Culicoides* capture sites used in our entomological survey.

In detail, adult *Culicoides* were collected using Onderstepoort-type 220 V black light traps at eight different locations, covering the entire region (Figure 1). Traps were positioned about 1.8 m above the ground, outside shelters and close to the livestock (mainly sheep). *Culicoides* were caught weekly, operating overnight from dusk until dawn of the following day, from May to December 2021.

*Culicoides* caught in the field were sent to the laboratory for counting and morphological identification, using appropriate morphological identification keys [29,30,31]. All *Culicoides* were sexed, and females were differentiated following the criteria of Dyce et al. [32] into nulliparous (unpigmented that had no blood meal), parous (pigmented that had at least one blood meal), gravid (that had well-formed eggs in their abdomen), and engorged (that had fresh blood in their abdomen). Only parous, gravid, and engorged females were subjected to real-time RT-PCR analysis to detect SBV, as only they had consumed at least one blood meal and could have ingested the virus.

Before being stored at −80 °C, *Culicoides* were pooled with a maximum of 25 specimens per pool, according to species, abdominal pigmentation, site, and date of collection.

## 3. Results

### 3.1. Anatomo-Pathological Survey

The results of the retrospective survey, based on the anatomo-pathological lesions we observed during the diagnostic activity between 2013 and 2023 of our Institute, are summarized in Table 1.

The table shows that the number of SBV infections in newborn ruminants, as determined by the anatomo-pathological observations and RT-PCR analysis, were sporadic after 2013, without an epidemic wave being recorded. During these years, SBV RNA was detected only in five malformed newborn lambs and one kid.

### 3.2. Serological Survey

The results of serological testing in farms investigated for the occurrence of infertility and malformed newborns show that the SBV seropositivity varied within species and years (Table 2).

The geographical distribution of the randomly selected sheep, which serologically yielded positive results for SBV antibodies, is shown in Figure 1.

Seroprevalence in the years 2022 and 2024 varied between 16.40% (C.I. = 12.28–20.52) and 21.53% (C.I. = 17.15–25.91) (Table 3); no significative differences were evidenced between the seroprevalence in the two years analyzed (χ^2^ = 2.770, *p*-value = 0.096). Figure S2 shows the frequency distribution by age of the analyzed and positive samples.

### 3.3. Isolation of SBV in Cell Cultures

SBV was isolated from the meninges and thymus of one stillborn lamb 4884/IT|2021 on VERO cell culture and from the brain of a second stillborn lamb 15353/IT|2021 on BHK21 cell culture. No virus was isolated on both VERO and BHK21 cell cultures from the six insect pool samples of *Culicoides imicola*, which resulted positive for SBV RNA.

### 3.4. Sequencing

We obtained the S and M-segment sequences from the two above-mentioned SBV isolates, the S sequences from one positive sample detected in 2020, and twenty-one archived cDNA samples. All samples were collected from malformed lambs. Fourteen S sequences collected in 2012 were from different tissues of seven animals belonging to the same farm. The sequences were deposited in GenBank under the following accession numbers: PQ424227–PQ424229. Detailed information is presented in Table S2.

The partial S-segments obtained in this study, corresponding to the whole N protein ORF, were 702 bp long and showed 99.0–100% similarity among themselves and 99.4–99.8% similarity with the reference SBV strain BH80/11-4 (HE649914; Germany, 2011). Fourteen different haplotypes were evidenced between Sardinian S sequences. Figure S3 shows the amino acid alignment between the N and NSs protein sequences and the reference SBV strain BH80/11-4 (HE649914; Germany, 2011). Eight amino-acidic substitutions were detected both in the N and in the NSs protein, six of which were in sequences from samples collected in 2012. The Ala120Thr and Ile67Thr substitutions, which were found in the N and NSs proteins, respectively, are unique in the NCBI database. Furthermore, sample number 4884/IT|2021, a cell culture isolated from ovine brain (Table S2), showed a premature stop codon in position 65 of the NSs protein. Blast analysis showed that the Italian S-segment sequences had the highest similarity (99.57–100%) with strains from Hungary, Belgium, the United Kingdom, and Germany collected in 2012, 2014, and 2017, especially from malformed lambs but also from acutely infected cattle.

The M-segment sequences from the two SBV isolated in culture cells were 4212 bp long and showed 99.1% similarity among themselves and 99.3% similarity with the reference SBV strain BH80/11-4 (HE649913; Germany, 2011). Figure S4 shows amino acid alignment between the M sequences obtained in this study and the reference strain BH80/11-4. Overall, 29 amino acidic substitutions were detected, mostly in the mutation hot spot of the Gc protein, highlighted in red square in Figure S4.

Blast analysis showed that the Italian M sequences had the highest similarity (99.36%, 99.38%) with strains from the Netherlands, Switzerland, and Hungary isolated in 2011, 2012, and 2014, respectively, from bovine blood.

### 3.5. Phylogenetic Analysis

**Figure 2:**
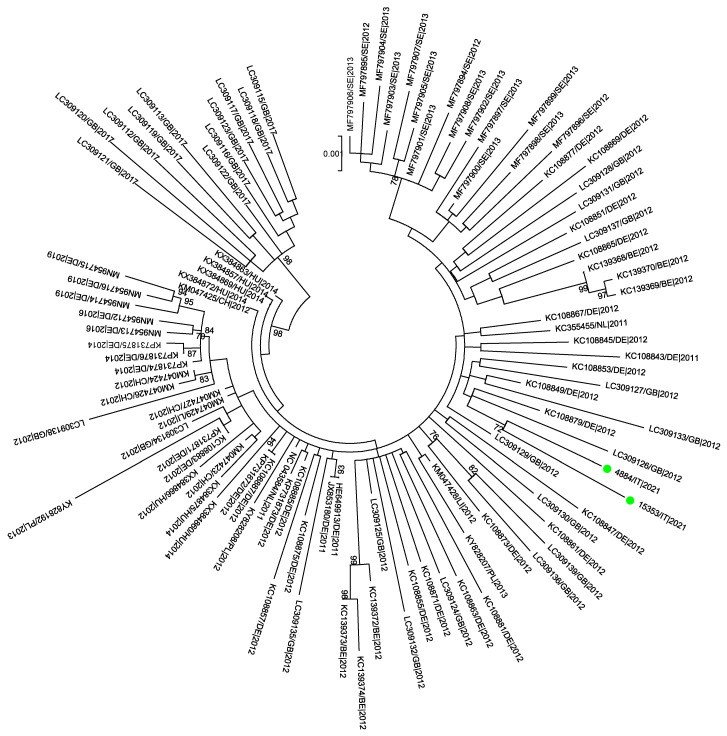
A maximum likelihood phylogenetic tree inferred from the S dataset composed of 160 SBV strains by the GTR+G+I model of nucleotide substitution. Sequences under study from recent SBV outbreaks are indicated with green circles. Bootstrap values < 70 are not shown. The scale bar indicates the number of substitutions per site.

**Figure 3:**
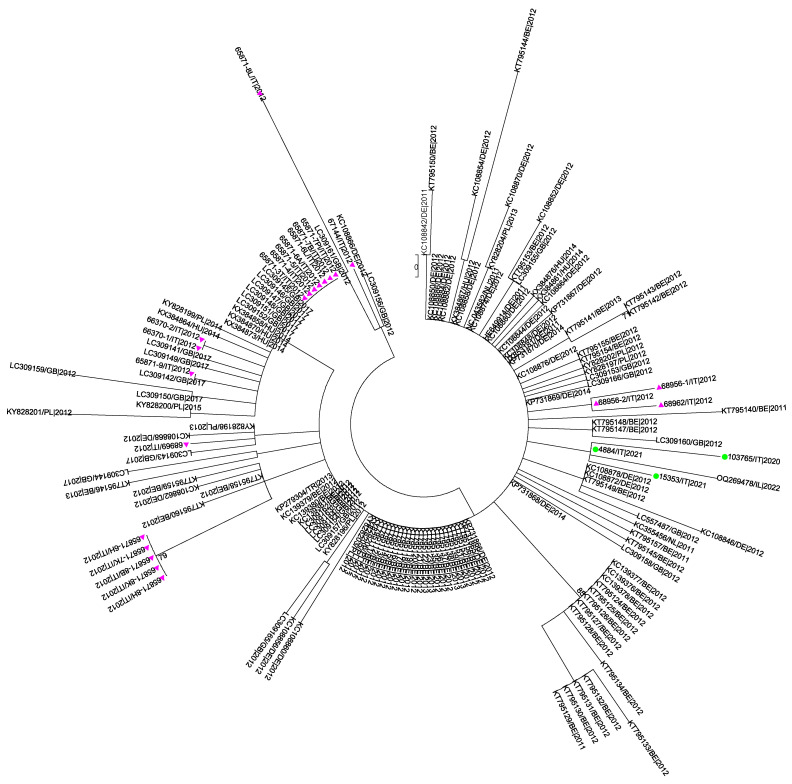
A maximum likelihood phylogenetic tree inferred from the M dataset composed of 105 SBV strains by the GTR+G+I model of nucleotide substitution. Isolates under study are indicated with green circles, and sequences from the epidemic wave in 2012 are indicated in purple triangles. Bootstrap values < 70 are not shown. The scale bar indicates the number of substitutions per site.

The recombinant analysis performed using RDP5 showed no evidence of recombination between the sequences included in the datasets analyzed. The phylogenetic signals, depicted in Figure S5, evidenced sufficient phylogenetic information only for the dataset M. The phylogenetic trees obtained from the datasets S and M are shown in Figure 2 and Figure 3.

As expected, given the high similarity between the S-segment sequences and the insufficient phylogenetic signal, low bootstrap values were evidenced in the phylogenetic tree based on the S dataset. The Sardinian S sequences were distributed in different branches of the tree, but only a cluster composed of five old Sardinian sequences from the same farm showed a good bootstrap value (79%).

The tree constructed on the M-segment sequences showed that the two Sardinian strains clustered together with a good statistical support (bootstrap 72%) within an unresolved cluster grouping strains from different geographical areas collected in the years 2011–2013.

### 3.6. Entomological Survey

From May to December 2021, a total of 226 catches were made at eight locations, yielding a total of 133,711 *Culicoides* belonging to 19 species (Table 4).

The most common species were *C. imicola* with 74,661 and *C. newsteadi* with 47,215 specimens, accounting for over 90% of the total. The other species were caught in much smaller numbers (Table 4).

**Figure 4:**
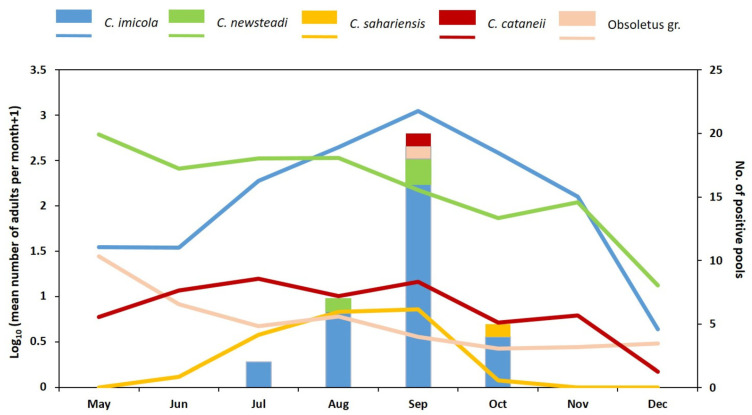
Seasonal abundance of *Culicoides* species found SBV positive (lines) and number of positive pools per month (bars).

Out of 1887 *Culicoides* pools, 34 tested positive for SBV by qRT-PCR. Among these positive pools, 29 comprised parous females and 5 comprised gravid females, while none of the pools of engorged females tested positive (Table S3). The most prevalent species was *C. imicola*, identified in 28 positive pools; each of these pools contained between 9 and 25 specimens and had Ct values ranging from 26.67 to 38.10. This was followed by *C. newsteadi*, detected in 3 pools (each with 9 to 25 specimens and Ct values ranging from 26.67 to 38.10). Additionally, one pool each of *C. cataneii* (4 specimens, Ct value = 36.38), *C. sahariensis* (2 specimens, Ct value = 36.00), and Obsoletus gr. (3 specimens, Ct value = 36.80) tested positive. SBV RNA was detected at least once at every capture site from July through October. Figure 4 shows the population trend of the SBV RNA-positive *Culicoides* species.

In particular, *C. imicola* was caught throughout the study period, with a significant population peak in September, while *C. newsteadi* showed higher densities from May to August (Figure 4).

## 4. Discussion

The review of diagnostic data from the identification of typical malformations in newborns and real-time PCR analysis suggest that SBV circulated enzootically in the Sardinian ruminant population over the years following the first epidemic wave in 2012. Moreover, serological data and virological examination of captured *Culicoides* indicate one widespread and homogenous distribution of the virus across the island.

Regarding the impact of this presence, SBV infection in Sardinian livestock appears to cause sporadic occurrence of malformations in newborn ruminants, often without detectable presence of viral RNA. This phenomenon is not surprising and has been explained by considering that SBV induces initial lesions in the fetus at an early stage of gestation, rendering the virus undetectable in the newborn [20].

However, given the nature of our data, which should be primarily considered the result of passive surveillance, and the reluctance of farmers to report the disease, it cannot be ruled out that a significant proportion of SBV infections has gone undetected.

Additionally, the low number of PCR and the cell culture positive samples should be considered, along with the fact that anatomo-pathological changes in newborn lambs are non-specific and may be associated with various genetic defects [33], toxico-metabolic causes [34], and virus infections [35].

Thus, it remains difficult to assess the exact numerical impact of SBV infections, although the frequency of these malformations in newborn ruminants was considerable higher than that recorded in the diagnostic activity of our institute in the years preceding the epidemic emergence of this virus. In this regard, only sporadic genetic malformations were reported in the years before 2012, with none attributed to a viral etiology.

When studying the dynamics of SBV circulation by recording the number of congenital malformations or detecting differences in seroprevalence over the years, it was observed that this virus is significantly more prevalent in certain years, with systematically larger epidemic waves hypothesized to occur approximately every 3 years [36]. This phenomenon is likely due to the replacement of previously exposed ruminant populations, resulting in a naturally lower herd immunity [37,38].

Our retrospective anatomo-pathological study did not reveal significant peaks of SBV circulation in Sardinia over the years, despite the virus being constantly present, establishing an endemic and widespread presence. The estimated seroprevalences in the years 2022 and 2024 are not significantly different and are lower than those recently reported in cattle and water buffalo in Southern Italy [5] and in wild ruminants in Germany [39].

In Sardinia, flocks breeding is seasonally regulated according to sheep age. Adult pluriparous ewes are mated in June/July, with lambing occurring in November/December, whereas nulliparous ewe lambs, the flock replacements, are mated in October/November with the lambing in February/March. Consequently, the naive ewe lamb of the flock are not pregnant when exposed to SBV as *Culicoides* populations increase in August and September. These ewe lambs become naturally immunized before pregnancy when SBV circulates, thereby protecting the fetus from infection. Since the resurgence of new epidemic waves of SBV requires a high proportion of naïve susceptible animals [40], we hypothesize that Sardinian reproductive management practices contribute to immunizing the naïve population before gestation. In SBV-infected cattle and sheep, anti-SBV antibodies persist for at least 38 and 48 months, respectively [41], although in naturally infected cattle, they may last up to six years [42].

Our study identified a high *Culicoides* species diversity, with 19 species recorded. However, since only parous, gravid and engorged females were examined, RT PCR analysis was conducted on only 16 species. Five species tested SBV positive: *C. imicola*, *C. newsteadi*, *C. cataneii*, *C. sahariensis* and the species within the *Obsoletus* group. Following the first Schmallenberg outbreak in 2011, *C. imicola*, *C. newsteadi,* and the *Obsoletus* group tested positive for SBV in several European countries [8,9,10,15,43,44]. These last three species are currently considered important vectors of SBV in Europe [45,46]. Concerning seasonal distribution, the highest number of positive pools was recorded between August and October, peaking in September, with a similar trend observed in Belgium, France, and Poland [8,10,11].

In 2012, a study conducted partly in Sardinia identified a low detection rate of SBV RNA exclusively in *C. imicola* specimens. However, it should be noted that this study involved only 26 *Culicoides* catches during the late vector season [18]. In our study, we find two new SBV-positive species, *C. cataneii* and *C. sahariensis*, which have not previously been associated with the transmission of this arbovirus. Notable, *C. cataneii* was the fifth most abundant species detected across all the surveyed sites and, although considered an ornithophilic species [47], it is frequently captured in Sardinia using light traps placed near livestock [48,49]. *Culicoides sahariensis* was caught at all sites but in low numbers, ranking as the ninth most abundant species. Limited information is available regarding the biology of this species. In Saudi Arabia, *C. sahariensis* specimens were caught while biting camels and donkeys [50,51]. Previous studies indicate that this species does not reach high population densities in Sardinia [48,49].

Despite the recommendations provided by Veronesi et al. [52] regarding vector competence, determining whether positive specimens are fully disseminated and thus competent for virus transmission remains challenging. Although these two species were found in low density, the widespread presence of SBV RNA in livestock farms in certain Mediterranean countries suggests they may act as potential vectors. Further studies are necessary to evaluate their vector competence for SBV and investigate the potential involvement of other *Culicoides* species.

In the present study, the S-segment sequences from SBV-positive samples collected in 2020–2021 and archived cDNA samples from malformed lambs during the first epidemic episode in 2012 were obtained. Phylogenetic analysis indicated that Sardinian strains belong to different branches without statistical significance. However, the presence of several substitutions in both the N and the NSs protein, two of which (N protein: Ala120Thr, NSs: Ile67Thr) are specific to Sardinian sequences, suggests a local viral evolution.

A total of 12 out of 24 sequences, regardless of isolation year, exhibited the amino acid substitution S111N in the N protein (nucleotide exchange G363A) that has been frequently observed in malformed fetuses. Recently, this substitution was associated with the inability to replicate in KC cells without affecting replication in Vero and BHK-21 cells [48]. In viremic animals, only the 363G variant was observed, whereas in malformed fetal tissues, the G363A substitution was predominantly found [53].

Furthermore, the cell culture isolate 4884 exhibited a premature stop codon at position 65 of the NSs protein. This finding has been previously reported only in malformed fetuses [53,54]. The NSs protein inhibits the host–cell gene expression and interferon (IFN) induction, thereby suppressing innate immune responses. It also plays roles in translation regulation and apoptosis [55]. Its biological activity is linked to the C-terminus [54,55,56,57], suggesting that truncated NSs sequences may represent avirulent viruses [54]. In fetal brain tissue, where the interferon response is minimal, there is no selective pressure to maintain an intact NSs protein [53].

In this study, due to the limited cDNA quantity of archived samples, only the M-segment sequences from the two cell culture isolates were successfully obtained. The phylogenetic analysis revealed that the Sardinian strains are significantly distinct (bootstrap 72%) from international strains collected during both the first SBV epidemic wave and the following re-emergences. Alignment with the reference strain identified 29 amino acidic substitutions, predominally in the mutation hotspot within the 5′-terminal region of the M-segment, which encodes the major SBV envelope protein Gc [58]. This region has been characterized as being critical for SBV neutralization, and the high sequence variability observed in malformed newborns is believed to be associated with viral immune escape mechanisms [13]. However, as M-segment sequences derived from malformed fetuses are considered artefactual and not representative of circulating strains [13], any inferences regarding viral evolution should be interpreted with caution. In conclusion, unlike what has been observed in other countries [54], the results of the phylogenetic analysis do not support the hypothesis that the most recent strains are significantly different from those circulating in 2012.

Finally, anatomo-pathological, serological, virological, and entomological data indicate that SBV has circulated continuously in Sardinia over the last decade with minimal impact on the ruminant population. This suggests that the likelihood of SBV resurgence at a level comparable to its initial emergence in 2012 is very low.

## Figures and Tables

**Table 1:** Number of sheep, cattle, and goat farms with SBV cases occurred from 2013 to 2023 in Sardinia. A total number of 340 malformed ruminant newborns were examined by RT-PCR and/or anatomo-pathologically.

Years	Species	No. Examined Farms	No. Farms with Malformed Newborns	No. Farms with RT-PCR Positive Newborns
2013–2015	Sheep	91	35	0
	Cattle	20	5	0
	Goat	12	1	1
2016–2018	Sheep	49	12	1
	Cattle	4	0	0
	Goat	4	0	0
2019–2021	Sheep	9	4	3
	Cattle	2	0	0
	Goat	2	0	0
2022–2023	Sheep	11	8	1
	Cattle	7	0	0
	Goat	2	1	0

**Table 2:** Number of serum samples and farms examined for SBV antibodies from 2013 to 2023.

Year	Host	No. Tested Samples (% Positive)	No. Tested Farms (% Positive)
2013	sheep	7348 (33.89)	206 (79.61)
	cattle	1735 (69.74)	51 (92.16)
	goat	957 (29.15)	42 (85.71)
2014	sheep	3516 (27.93)	65 (87.69)
	cattle	1007 (90.37)	18 (88.89)
	goat	98 (19.39)	4 (N.A.)
2015	sheep	1623 (7.33)	47 (46.81)
	cattle	418 (25.84)	17 (76.47)
	goat	380 (10.00)	2 (N.A.)
2016	sheep	611 (7.36)	32 (28.13)
	cattle	613 (22.68)	12 (91.67)
	goat	421 (8.31)	9 (N.A.)
2017	sheep	109 (10.09)	23 (34.78)
	cattle	124 (46.77)	14 (64.29)
	goat	1 (0.00)	1 (N.A.)
2018	sheep	118 (7.63)	19 (26.32)
	cattle	104 (42.31)	19 (68.42)
	goat	48 (0.00)	2 (N.A.)
2019	sheep	13 (7.69)	3 (N.A.)
	cattle	55 (67.27)	6 (N.A.)
	goat	20 (15.00)	2 (N.A.)
2020	sheep	30 (13.33)	5 (N.A.)
	cattle	103 (36.89)	12 (83.33)
	goat	17 (11.76)	2 (N.A.)
2021	sheep	140 (48.57)	11 (54.55)
	cattle	102 (90.20)	15 (100)
2022	sheep	34 (8.82)	7 (N.A.)
	cattle	34 (64.71)	6 (N.A.)
	goat	2 (N.A.)	1 (N.A.)
2023	sheep	79 (2.53)	3 (N.A.)
	cattle	23 (95.65)	4 (N.A.)

N.A. = not applicable.

**Table 3:** SBV antibody detection during 2022 and 2024 in randomly selected Sardinian sheep (Italy).

Year	No. Sheep/% Positive	C.I. 95%
2022	311/16.40	12.28–20.52
2024	339/21.53	17.15–25.91

C.I. = confidence interval.

**Table 4:** Species and total number of the *Culicoides* captured in the 8 Sardinian municipalities from May to December 2021.

Species	Municipalities							
	Alghero (25)	Arborea (21)	Iglesias (31)	Palau (32)	Quartu S.E. (30)	S.A. Arresi (29)	Siniscola (30)	Villacidro (28)
*C. imicola*	1012	1449	8765	45,279	2266	1202	11,297	3391
*C. newsteadi*	1631	358	1847	36,238	106	391	5318	1326
*C. circumscriptus*	79	13	340	432	14	1710	245	236
*C. jumineri*	71	82	103	430	8	165	102	1662
*C. cataneii*	227	7	177	477	13	899	59	96
*Obsoletus gr.*	79	27	318	377	125	2	91	21
*C. kibunensis*	28	0	36	270	31	4	22	2
*C. punctatus*	37	41	67	198	6	28	192	293
*C. sahariensis*	22	1	85	168	2	160	14	49
*C. pulicaris*	333	0	4	82	10	0	3	7
*C. univittatus*	0	0	31	154	7	240	0	2
*C. paolae*	23	4	34	10	13	185	17	33
*C. festivipennis*	32	0	13	33	2	0	1	0
*C. puncticollis*	27	0	1	4	0	29	4	0
*C. maritimus*	0	3	0	0	0	43	3	0
*C. parroti*	2	0	0	0	0	0	0	0
*C. subfagineus*	0	0	1	0	0	0	0	0
*C. brunnicans*	1	0	0	0	0	0	0	0
*C. lupicaris*	1	0	0	0	0	0	0	0
Total	3605	1985	11,822	84,152	2603	5058	17,368	7118

## Data Availability

The original contributions presented in this study are included in the article/Supplementary Materials. Further inquiries can be directed to the corresponding author.

## Supplementary Materials

The following supporting information can be downloaded at https://www.mdpi.com/article/10.3390/pathogens14040349/s1. Figure S1: Stillborn lambs affected by SBV. Congenital malformations include severe arthrogryposis of all four limbs, kyphosis, and torticollis (a–e), brachygnathia of the lower jaw (f), cerebellar hypoplasia (black arrow) (g), hydrocephalus (h), and unilateral hydronephrosis (i). Figure S2: SBV antibody detection in 2022 (a) and 2024 (b) in randomly selected Sardinian sheep (Italy). The graphs show the frequency distribution by age of the analyzed (in blue) and positive samples (in red). Figure S3: Amino acid alignment of the Italian N (a) and NSs (b) SBV sequences with BH80/11-4 reference strain. Figure S4: Amino acid alignment between the M sequences obtained in this study and the reference strain BH80/11-4. The mutation hot spot of the Gc protein is highlighted in red. Figure S5: Phylogenetic signal of the datasets analyzed in this study. a: Dataset S; b: dataset M. Table S1: List of primers used for the sequencing of SBV S and M-segments. Table S2: The year of collection, host, source, municipalities, SBV segment, and GenBank accession number of the SBV strains analyzed in this study. Table S3: Number of pool positive, pool analyzed, and total analyzed *Culicoides* adults.
