# Supplementary material for: Assessing Schmallenberg Virus Disease in Sardinia (Italy) After the First Epidemic Episode in 2012
Source: Pathogens. 2025 Apr 4;14(4):349. doi: 10.3390/pathogens14040349 (PMC12030605; doi:10.3390/pathogens14040349)
Supplement: Supplementary file 1 [file pathogens-14-00349-s001.zip › Table S2.pdf]

Table S2. The year of collection, host, source, municipalities, SBV segment and GenBank accession number of the SBV strains analysed in this study.

Strain	Year	Host	Source	Municipality/ Province	SBV Segment	Accession Number
65871-3T/IT|2012	2012	Sheep	Tymus	Mores/SS	S	PQ424204
65871-4/IT|2012	2012	Sheep	Brain	Mores/SS	S	PQ424205
65871-5/IT|2012	2012	Sheep	Brain	Mores/SS	S	PQ424206
65871-6A/IT|2012	2012	Sheep	Abomasum	Mores/SS	S	PQ424207
65871-6H/IT|2012	2012	Sheep	Hearth	Mores/SS	S	PQ424208
65871-6L/IT|2012	2012	Sheep	Lung	Mores/SS	S	PQ424209
65871-7B/IT|2012	2012	Sheep	Brain	Mores/SS	S	PQ424210
65871-7Pl/IT|2012	2012	Sheep	Placenta	Mores/SS	S	PQ424211
65871-7K/IT|2012	2012	Sheep	Kidney	Mores/SS	S	PQ424212
65871-8B/IT|2012	2012	Sheep	Brain	Mores/SS	S	PQ424213
65871-8H/IT|2012	2012	Sheep	Hearth	Mores/SS	S	PQ424214
65871-8L/IT|2012	2012	Sheep	Lung	Mores/SS	S	PQ424215
65871-8K/IT|2012	2012	Sheep	Kidney	Mores/SS	S	PQ424216
65871-9/IT|2012	2012	Sheep	Brain	Mores/SS	S	PQ424217
66370-1/IT|2012	2012	Sheep	Brain	Ozieri/SS	S	PQ424218
66370-2/IT|2012	2012	Sheep	Lung	Ozieri/SS	S	PQ424219
67144/IT|2012	2012	Sheep	Brain	Sassari/SS	S	PQ424220
68956-1/IT|2012	2012	Sheep	Brain	Olbia/OT	S	PQ424221
68956-2/IT|2012	2012	Sheep	Fleece	Olbia/OT	S	PQ424222
68962/IT|2012	2012	Sheep	Brain	Olbia/OT	S	PQ424223
68969/IT|2012	2012	Sheep	Brain	Olbia/OT	S	PQ424224
103765/IT|2020	2020	Sheep	Brain	Ittiri/SS	S	PQ424225
4884/IT|2021	2021	Sheep	Isolate from brain	Uras/OR	S<br>M	PQ424226<br>PQ424228
15353/IT|2021	2021	Sheep	Isolate from brain	Paulilatino/OR	S<br>M	PQ424227<br>PQ424229
