# Supplementary material for: Assessing Schmallenberg Virus Disease in Sardinia (Italy) After the First Epidemic Episode in 2012
Source: Pathogens. 2025 Apr 4;14(4):349. doi: 10.3390/pathogens14040349 (PMC12030605; doi:10.3390/pathogens14040349)
Supplement: Supplementary file 1 [file pathogens-14-00349-s001.zip › Table S3.pdf]

**Table S3**. Number of pool positive, pool analysed and total of analized *Culicoides* adults

| Species | Alghero | Arborea | Iglesias | Palau | Quartu S. E. | S. A. Arresi | Siniscola | Villacidro | **Total** |
|---|---|---|---|---|---|---|---|---|---|
| *C. imicola* | **2**/52/848 | **4**/38/402 | **5**/147/2947 | 0/113/1991 | **5**/67/972 | **1**/61/803 | **8**/138/2742 | **3**/82/1374 | **28/698/12079** |
| *C. newsteadi* | 0/51/934 | 0/22/199 | **1**/60/963 | **1**/127/2458 | **1**/9/42 | 0/32/202 | 0/119/2465 | 0/30/342 | **3/460/7605** |
| *C. circumscriptus* | 0/8/54 | 0/6/6 | 0/29/171 | 0/23/69 | 0/2/2 | 0/53/689 | 0/16/137 | 0/25/153 | **0/162/1281** |
| *C. cataneii* | **1**/20/206 | 0/5/7 | 0/19/84 | 0/24/95 | 0/4/4 | 0/48/619 | 0/9/30 | 0/17/51 | **1/146/1096** |
| *C. jumineri* | 0/10/37 | 0/8/24 | 0/14/29 | 0/12/28 | 0/2/2 | 0/20/100 | 0/5/11 | 0/27/251 | **0/98/482** |
| *C. punctatus* | 0/9/28 | 0/5/23 | 0/7/43 | 0/8/16 | 0/1/2 | 0/5/9 | 0/13/157 | 0/11/90 | **0/59/368** |
| Obsoletus gr. | 0/9/30 | 0/4/10 | **1**/20/120 | 0/12/26 | 0/19/61 | 0/1/1 | 0/12/42 | 0/6/7 | **1/83/297** |
| *C. sahariensis* | **1**/4/20 | 0/1/1 | 0/11/44 | 0/8/21 | | 0/13/122 | 0/3/6 | 0/9/37 | **1/49/251** |
| *C. univittatus* | | | 0/3/7 | 0/5/48 | | 0/13/105 | | | **0/21/160** |
| *C. pulicaris* | 0/15/122 | | 0/3/4 | 0/5/9 | 0/3/5 | | 0/1/1 | 0/1/1 | **0/28/142** |
| *C. paolae* | 0/5/8 | 0/2/2 | 0/7/17 | **1**/1/1 | 0/1/1 | 0/16/58 | 0/1/2 | 0/3/4 | **1/36/93** |
| *C. festivipennis* | 0/5/30 | | 0/3/5 | 0/1/2 | | | | | **0/9/37** |
| *C. kibunensis* | 0/4/11 | | 0/2/2 | 0/4/6 | 0/1/6 | 0/1/4 | 0/1/1 | | **0/13/30** |
| *C. maritimus* | | 0/1/1 | | | | 0/7/28 | 0/1/2 | | **0/9/31** |
| *C. puncticollis* | 0/4/9 | | | 0/1/1 | | 0/8/15 | 0/2/3 | | **0/15/28** |
| *C. parroti* | 0/1/1 | | | | | | | | **0/1/1** |
| **Total** | **4/197/2338** | **4/92/675** | **7/325/4436** | **2/344/4771** | **6/119/1097** | **1/278/2755** | **8/321/5509** | **3/211/2310** | **35/1887/23981** |
