# Supplementary material for: Assessing Schmallenberg Virus Disease in Sardinia (Italy) After the First Epidemic Episode in 2012
Source: Pathogens. 2025 Apr 4;14(4):349. doi: 10.3390/pathogens14040349 (PMC12030605; doi:10.3390/pathogens14040349)
Supplement: Supplementary file 1 [file pathogens-14-00349-s001.zip › Figure S2.pdf]

a

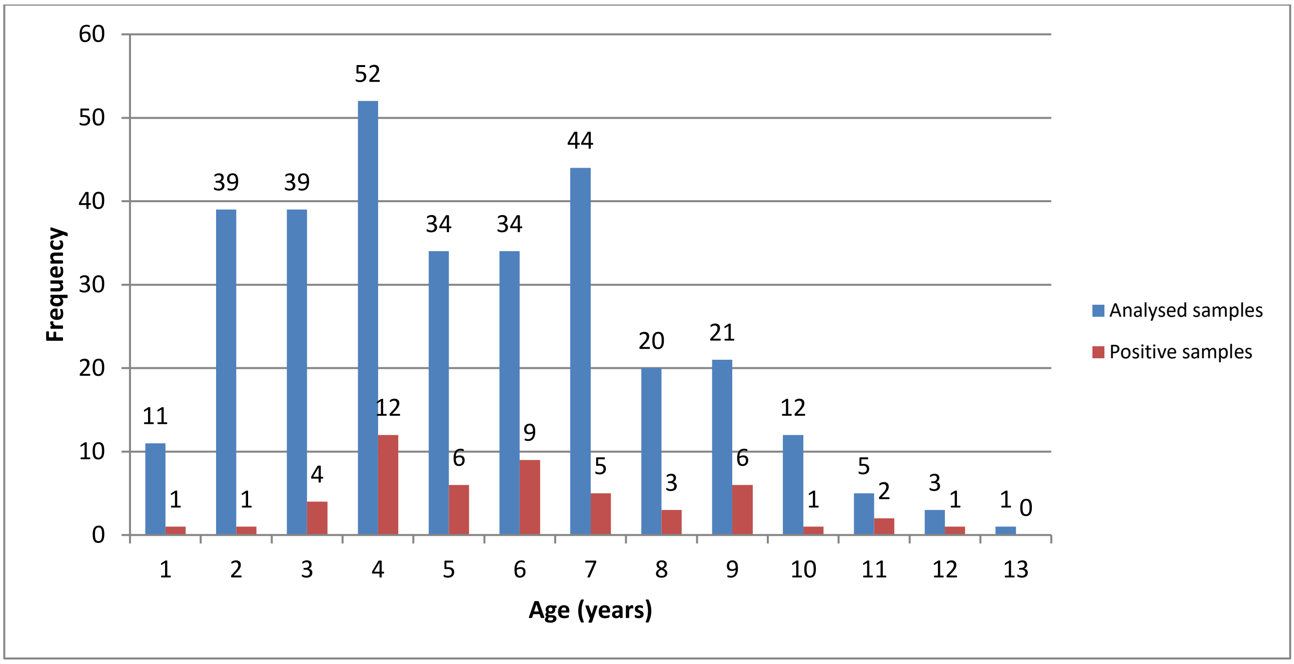

b

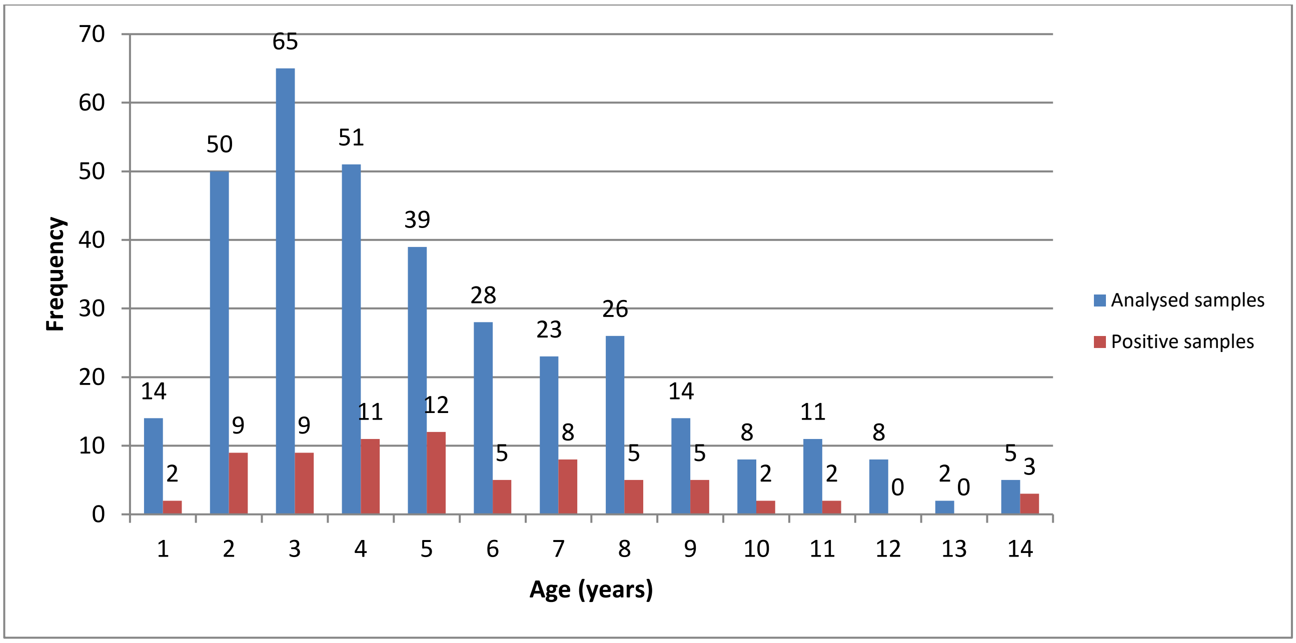

Figure S2: SBV antibody detection during 2022 (a) and 2024(b) in randomly selected Sardinian sheep (Italy). The graphs show the frequency distribution by age of the analysed (in blue) and positive samples (in red).
