# Supplementary material for: Assessing Schmallenberg Virus Disease in Sardinia (Italy) After the First Epidemic Episode in 2012
Source: Pathogens. 2025 Apr 4;14(4):349. doi: 10.3390/pathogens14040349 (PMC12030605; doi:10.3390/pathogens14040349)
Supplement: Supplementary file 1 [file pathogens-14-00349-s001.zip › Figure S3.pdf]

a

```
HE649914/BH80/11-4/DE|2011  1   MSSQFIFEDV PQRNAATFNP EVGYVAFIGK YGQQLNFGVA RVFFLNQKKA KMVLHKTAQP SVDLTFGGVK FTVVNNHFPQ YVSNPVPDNA ITLHRMSGYL 100
65871-3T/IT|2012            1   .......... .......... .......... .......... .......... .......... .......... .......... .......... .......... 100
65871-4/IT|2012             1   .......... .......... .......... .......... .......... .......... .......... .......... .......... .......... 100
65871-5/IT|2012             1   .......... .......... .......... .......... .......... .......... .......... .......... .......... .......... 100
65871-6A/IT|2012            1   .......... .......... .......... .......... .......... .......... .......... .......... .......... .......... 100
65871-6H/IT|2012            1   .......... .......... ........S. .......... .......... .......... .......... .......... .......... .......... 100
65871-6L/IT|2012            1   .......... .......... .......... .......... .......... .......... .......... .......... .......... .......... 100
65871-7B/IT|2012            1   .......... .......... .......... .......... .......... .......... .......... .......... .......... .......... 100
65871-7P1/IT|2012           1   .......... .......... .......... .......... .......... .......... .......... .......... .......... .......... 100
65871-7K/IT|2012            1   .......... .......... ........S. .......... .......... .......... .......... .......... .......... .......... 100
65871-8B/IT|2012            1   .......... .......... ........S. .......... .......... .......... .......... .......... .......... .......... 100
65871-8H/IT|2012            1   .......... .......... ........S. .......... .......... .......... .......... .......... .......... .......... 100
65871-8L/IT|2012            1   .......... .......... .......... .......... .......... .......... .......... .......... .......... .......... 100
65871-8K/IT|2012            1   .......... .......... ........S. .......... .......... .......... .......... .......... .......... .......... 100
65871-9/IT|2012             1   .......... .......... .......... .......... .......... .......... .A........ .......... .......... .......... 100
66370-1/IT|2012             1   .......... .......... .......... .......... .......... .......... .......... .......... .......... .......... 100
66370-2/IT|2012             1   .......... .......... .......... .......... .......... .......... .......... .......... .......... .......... 100
67144/IT|2012               1   .......... .......... .......... .......... .......... .......... .......... .......... .......... .......... 100
68956-1/IT|2012             1   .......... .......... .......... .......... .......... .......... .......... .......... .......... .......... 100
68956-2/IT|2012             1   .......... .......... .......... .......... .......... .......... .......... .......... .......... .......... 100
68962/IT|2012               1   .......... .......... .......... .......... .......... .......... .......... .......... .......... .......... 100
68969/IT|2012               1   .......... .......... ........A. .......... .......... .......... .......... .......... .......... .......... 100
103765/IT|2020              1   .......... .......... .......... .......... .......... .......... .......... .......... .......... .......... 100
4884/IT|2021                1   .......... .......... .......... .......... .......... .......... .......... ...I...... .......... .......... 100
15353/IT|2021               1   .......... .......... .......... .......... .......... .......... .......... .......... .......... .......... 100

HE649914/BH80/11-4/DE|2011  101 ARWIADTCKA SVLKLAEASA QIVMPLAEVK GCTWADGYTM YLGFAPGAEM FLDAFDFYPL VIEMHRVLKD NMDVNFMKKV LRQRYGTMTA EEWMTQKITE 200
65871-3T/IT|2012            101 .......... .......... .......... .......... .......... .......... .......... .......... .......... .......... 200
65871-4/IT|2012             101 .......... N......... .......... .......... .......... .......... .......... .......... .......... .......... 200
65871-5/IT|2012             101 .......... .......... .......... .......... .......... .......... .......... .......... .......... .......... 200
65871-6A/IT|2012            101 .......... .......... .......... .......... .......... .......... .......... .......... .......... .......... 200
65871-6H/IT|2012            101 ...T...... .......... .......... .......... .......... .......... .......... .......... .......... .......... 200
65871-6L/IT|2012            101 .......... .......... .......... .......... .......... .......... .......... .......... .......... .......... 200
65871-7B/IT|2012            101 .......... N......... .......... .......... .......... .......... .......... .......... .......... .......... 200
65871-7P1/IT|2012           101 .......... N......... .......... .......... .......... .......... .......... .......... .......... .......... 200
65871-7K/IT|2012            101 ...T...... .......... .......... .......... .......... .......... .......... .......... .......... .......... 200
65871-8B/IT|2012            101 ...T...... .......... .......... .......... .......... .......... .......... .......... .......... .......... 200
65871-8H/IT|2012            101 ...T...... .......... .......... .......... .......... .......... .......... .......... .......... .......... 200
65871-8L/IT|2012            101 .......... NI........ .......... .......... .......... .......... .......... .......... .......... .......... 200
65871-8K/IT|2012            101 ...T...... .......... .......... .......... .......... .......... .......... .......... .......... .......... 200
65871-9/IT|2012             101 .......... N......... .......... .......... .......... .......... .......... .......... .......... .......... 200
66370-1/IT|2012             101 .......... N......... .......... .......... .......... .......... .......... .......... .......... .......... 200
66370-2/IT|2012             101 .......... N......... .......... .......... .......... .......... .......... .......... .......... .......... 200
67144/IT|2012               101 .......... N......... .......... .......... .......... .......... .......... .......... .......... .......... 200
68956-1/IT|2012             101 .......... .........T .......... .......... .......... .......... .......... .......... .......... .......... 200
68956-2/IT|2012             101 .......... .......... .......... .......... .......... .......... .......... .......... .......... .......... 200
68962/IT|2012               101 .......... .......... .......... .......... .......... .......... .......... .......... .......... .......... 200
68969/IT|2012               101 .......... N......... .......... .......... .......... .......... .......... .......... .......... .......... 200
103765/IT|2020              101 .......... N......... .......... .......... .......... .......... .......... .......... .......... .......... 200
4884/IT|2021                101 .......... N......... .......... .......... .......... .......... .......... .......... .......... .......... 200
15353/IT|2021               101 .......... N......... .......... .......... .......... .......... .......... .......... .......... .......... 200

HE649914/BH80/11-4/DE|2011  201 IKAAFNSVGQ LAWAKSGFSP AARTFLQQFG INI* 234
65871-3T/IT|2012            201 .......... .......... .......... ...* 234
65871-4/IT|2012             201 .......... .......... .......... ...* 234
65871-5/IT|2012             201 .......... .......... .......... ...* 234
65871-6A/IT|2012            201 .......... .......... .......... ...* 234
65871-6H/IT|2012            201 .......... .......... .......... ...* 234
65871-6L/IT|2012            201 .......... .......... .......... ...* 234
65871-7B/IT|2012            201 .......... .......... .......... ...* 234
65871-7P1/IT|2012           201 .......... .......... .......... ...* 234
65871-7K/IT|2012            201 .......... .......... .......... ...* 234
65871-8B/IT|2012            201 .......... .......... .......... ...* 234
65871-8H/IT|2012            201 .......... .......... .......... ...* 234
65871-8L/IT|2012            201 .......... .......... .......... ...* 234
65871-8K/IT|2012            201 .......... .......... .......... ...* 234
65871-9/IT|2012             201 .......... .......... .......... ...* 234
66370-1/IT|2012             201 .......... .......... .......... ...* 234
66370-2/IT|2012             201 .......... .......... .......... ...* 234
67144/IT|2012               201 .......... .......... .......... ...* 234
68956-1/IT|2012             201 .......... .......... .......... ...* 234
68956-2/IT|2012             201 .......... .......... .......... ...* 234
68962/IT|2012               201 .......... .......... .......... ...* 234
68969/IT|2012               201 .......... .......... .......... ...* 234
103765/IT|2020              201 .......... .......... .......... ...* 234
4884/IT|2021                201 .......... .......... .......... ...* 234
15353/IT|2021               201 .......... .......... .......... ...* 234
```

b

```
HE649914/BH80/11-4/DE|2011  1  MYHNGMQLHL TRRSGMWHLL VSMGNNSTSV LLESSSSTRR RPRWSYIRRH NQVSILLLVG SNLQWLITIF PNMSQILCQT MPLHFTGCQD I* 92
65871-3T/IT|2012            1  .......... .......... .......... .......... .......... .......... .......... .......... ...R...... .* 92
65871-4/IT|2012             1  .......... .......... .......... .......... .......... .......... .......... .......... ...R...... .* 92
65871-5/IT|2012             1  .......... .......... .......... .......... .......... .......... .......... .......... ...R...... .* 92
65871-6A/IT|2012            1  .......... .......... .......... .......... .......... .......... .......... .......... ...R...... .* 92
65871-6H/IT|2012            1  .......... .......... .......... .......... .......... .......... .......... .......... ...R...... .* 92
65871-6L/IT|2012            1  .......... .......... .......... .......... .......... .......... .......... .......... ...R...... .* 92
65871-7B/IT|2012            1  .......... .......... .......... .......... .......... .......... .......... .......... ...R...... .* 92
65871-7P1/IT|2012           1  .......... .......... .......... .......... .......... .......... .......... .......... ...R...... .* 92
65871-7K/IT|2012            1  .......... .......... .......... .......... .......... .......... .......... .......... ...R...... .* 92
65871-8B/IT|2012            1  .......... .......... .......... .......... .......... .......... .......... .......... ...R...... .* 92
65871-8H/IT|2012            1  .......... .......... .......... .......... .......... .......... .......... .......... ...R...... .* 92
65871-8L/IT|2012            1  .......... .......... .......... .......... .......... .......... .......... .......... .......... .* 92
65871-8K/IT|2012            1  .......... .......... .......... .......... .......... .......... .......... .......... ...R...... .* 92
65871-9/IT|2012             1  .......... .......... .......... .......... .......... ...P...... .......... .......... ...R...... .* 92
66370-1/IT|2012             1  .......... .......... .......... .......... .......... .......... .......... .......... ...R...... .* 92
66370-2/IT|2012             1  .......... .......... .......... .......... .......... .......... .......... .......... ...R...... .* 92
67144/IT|2012               1  .......... .......... .......... .......... .......... .......... .......... .......... ...R...... .* 92
68956-1/IT|2012             1  .......... .......... .......... .......... .......... .......... .......... .......... .......... .* 92
68956-2/IT|2012             1  .......... .......... .......... .......... .......... .......... .......... .......... .......... .* 92
68962/IT|2012               1  .......... .......... .......... .......... .......... .......... ......T... .......... .......... .* 92
68969/IT|2012               1  .......... .......... L......... .......... .......... .....S.... .......... .......... .......... .* 92
103765/IT|2020              1  ..R....... .......... .......... .......... .......... .......... .......... .......... .......... .* 92
4884/IT|2021                1  .......... .......... .......... .......... .......... .......... ....*..... .......... .......... .* 92
15353/IT|2021               1  .......... .......... .......... .......... .......... .......... .........S .......... .......... .* 92
```

Figure S3: Amino acid alignment of the Italian N (a) and NSs(b) SBV sequences with BH80/11-4 reference strain
