# Supplementary material for: Assessing Schmallenberg Virus Disease in Sardinia (Italy) After the First Epidemic Episode in 2012
Source: Pathogens. 2025 Apr 4;14(4):349. doi: 10.3390/pathogens14040349 (PMC12030605; doi:10.3390/pathogens14040349)
Supplement: Supplementary file 1 [file pathogens-14-00349-s001.zip › Figure S5.pptx]

## Slide 1
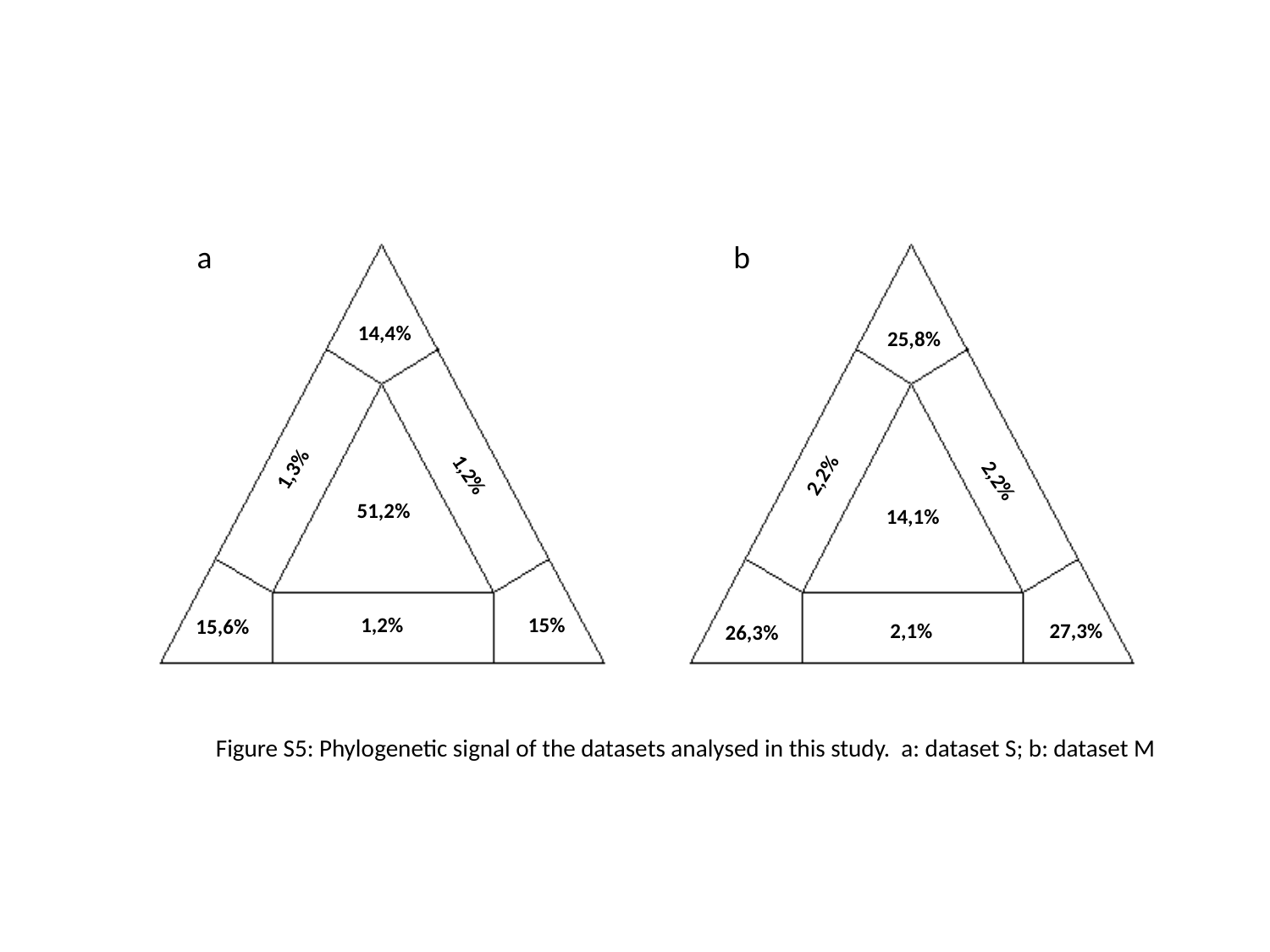

14,4%
1,3%
1,2%
51,2%
15%
1,2%
15,6%
a
25,8%
2,2%
2,2%
14,1%
27,3%
2,1%
26,3%
b
 Figure S5: Phylogenetic signal of the datasets analysed in this study. a: dataset S; b: dataset M
