# Supplementary material for: Assessing Schmallenberg Virus Disease in Sardinia (Italy) After the First Epidemic Episode in 2012
Source: Pathogens. 2025 Apr 4;14(4):349. doi: 10.3390/pathogens14040349 (PMC12030605; doi:10.3390/pathogens14040349)
Supplement: Supplementary file 1 [file pathogens-14-00349-s001.zip › Figure S1.pptx]

## Slide 1
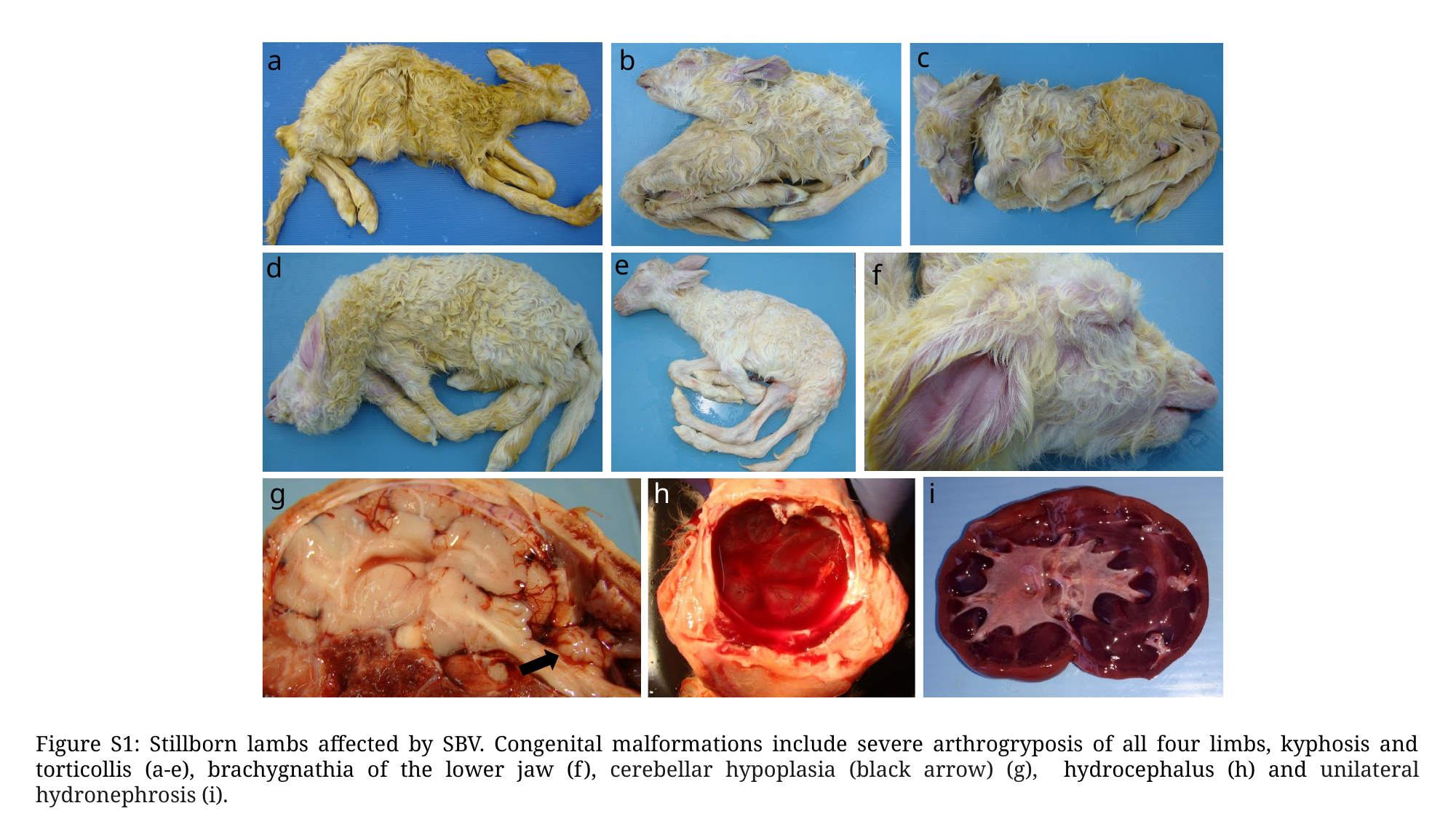

c
a
b
e
d
f
g
h
i
Figure S1: Stillborn lambs affected by SBV. Congenital malformations include severe arthrogryposis of all four limbs, kyphosis and torticollis (a-e), brachygnathia of the lower jaw (f), cerebellar hypoplasia (black arrow) (g), hydrocephalus (h) and unilateral hydronephrosis (i).
