# Supplementary material for: Assessing Schmallenberg Virus Disease in Sardinia (Italy) After the First Epidemic Episode in 2012
Source: Pathogens. 2025 Apr 4;14(4):349. doi: 10.3390/pathogens14040349 (PMC12030605; doi:10.3390/pathogens14040349)
Supplement: Supplementary file 1 [file pathogens-14-00349-s001.zip › Figure S4.pptx]

## Slide 1
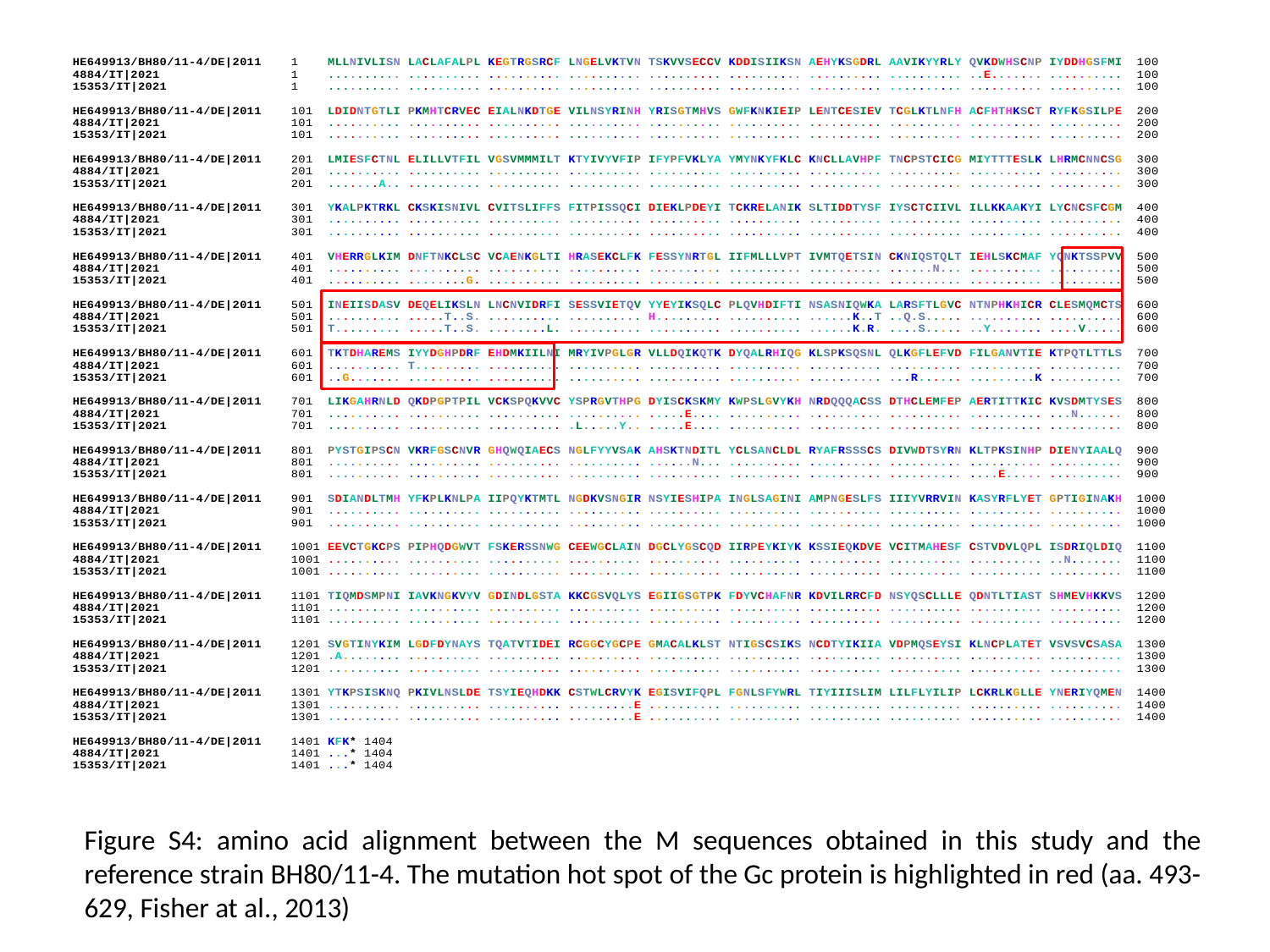

Figure S4: amino acid alignment between the M sequences obtained in this study and the reference strain BH80/11-4. The mutation hot spot of the Gc protein is highlighted in red (aa. 493-629, Fisher at al., 2013)
