# Supplementary material for: Assessing Schmallenberg Virus Disease in Sardinia (Italy) After the First Epidemic Episode in 2012
Source: Pathogens. 2025 Apr 4;14(4):349. doi: 10.3390/pathogens14040349 (PMC12030605; doi:10.3390/pathogens14040349)
Supplement: Supplementary file 1 [file pathogens-14-00349-s001.zip › Table S1.pdf]

Table S1. List of primers used for sequencing of SBV S and M-segments

Primer sequence	Amplicon length	Location	Reference
SBV-S_F*: GTGCTCCACTATTAACTACAGAAAT<br>SBV-S_R*: AGAAGCCTTGCAGTATAATGGTG	789	S-segment	*
SBV-M1_F: TGCTTCCTGAATGGCGAACT<br>SBV-M1_R: CGGGTGCATGACTTATGGGT	488	M-segment	This study
SBV-M2_F: CTACCGAATCTCGGGCACAA<br>SBV-M2_R: CACTTGGAGAGGGCACAACT	>1000	M-segment	This study
SBV-M3_F: GCAACTGCAGCTTTTGTGGT<br>SBV-M3_R: ATCATGCACTTGGAGAGGGC	491	M-segment	This study
SBV-M4_F: GCCCTCTCCAAGTGCATGAT<br>SBV-M4_R: ACGCAAGTCCAGGCAATTTG	935	M-segment	This study
SBV-M5_F: AGGAGCCCACAGAAACTTGG<br>SBV-M5_R: ACCTCTTCGTGCTTGGCATT	900	M-segment	This study
SBV-M6_F: AATGCCAAGCACGAAGAGGT<br>SBV-M6_R: TCAATTTGAGTGCGCAAGCC	757	M-segment	This study
SBV-M1_F*:AGTAGTGTACTACCACAATCA AAAT<br>SBV-M1_R: CGGGTGCATGACTTATGGGT	596	M-segment	*<br>This study
SBV-M6_F: AATGCCAAGCACGAAGAGGT<br>SBV-M6_R*: GTGAGTAGTGTTCTACCACATG	>1000	M-segment	This study<br>*

*Primer sequence kindly provided by Dr Martin Beer (FLI, Insel Riems)
